# Multiple-Locus Variable-Number Tandem-Repeat Analysis of Pathogenic *Yersinia enterocolitica* in China

**DOI:** 10.1371/journal.pone.0037309

**Published:** 2012-05-15

**Authors:** Xin Wang, Wenpeng Gu, Zhigang Cui, Longze Luo, Peng Cheng, Yuchun Xiao, Liuying Tang, Biao Kan, Huaiqi Jing

**Affiliations:** 1 National Institute for Communicable Disease Control and Prevention, Chinese Center for Disease Control and Prevention, State Key Laboratory for Infectious Disease Prevention and Control, Beijing, China; 2 Yunnan Provincial Centre for Disease Control and Prevention, Kunming, China; 3 Sichuan Provincial Centre for Disease Control and Prevention, Chengdu, China; 4 Huangshan Municipal Centre for Disease Control and Prevention, Huangshan, China; 5 National Institute for Viral Disease Control and Prevention, Chinese Center for Disease Control and Prevention, Beijing, China; Universidad Nacional de La Plata, United States of America

## Abstract

The predominant bioserotypes of pathogenic *Yersinia enterocolitica* in China are 2/O: 9 and 3/O: 3; no pathogenic O: 8 strains have been found to date. Multiple-Locus Variable-Number Tandem-Repeat Analysis (MLVA) based on seven loci was able to distinguish 104 genotypes among 218 pathogenic *Y. enterocolitica* isolates in China and from abroad, showing a high resolution. The major pathogenic serogroups in China, O: 3 and O: 9, were divided into two clusters based on MLVA genotyping. The different distribution of *Y. enterocolitica* MLVA genotypes maybe due to the recent dissemination of specific clones of 2/O: 9 and 3/O: 3 strains in China. MLVA was a helpful tool for bacterial pathogen surveillance and investigation of pathogenic *Y. enterocolitica* outbreaks.

## Introduction

*Yersinia enterocolitica* is a gram-negative entero-pathogen causing gastroenteritis, especially in children [1]. Human clinical infections with *Y. enterocolitica* occur after ingestion of contaminated food, water, or by direct inoculation through blood transfusion. Infection with *Y. enterocolitica* causes several clinical manifestations e.g. diarrhea, enteritis, enterocolitis, mesenteric lymphadenitis and some complications and sequelae, reactive arthritis, and erythema nodosum [2]. Lots of animal reservoirs are found for *Y. enterocolitica*, including swine, monkeys, dogs, rabbits, deer, and birds; swine is the predominant carrier [3]. Some research has shown high carrier rates of *Y. enterocolitica* in the swine oral cavity [4].

*Y. enterocolitica* is divided into six distinct biotypes and 60 serotypes. The biotypes of *Y. enterocolitica* are divided into three groups according to the bacteria's pathogenic properties: nonpathogenic biotype 1A, weakly pathogenic biotypes 2–5, and highly pathogenic biotype 1B [5]. Serotype O: 3, O: 8, and O: 9 strains have virulence factors and are pathogens commonly responsible for yersiniosis [6].

The distribution of pathogenic *Y. enterocolitica* is diverse in China and all strains belong to serotypes O: 3 and O: 9. These two serotypes can be isolated from livestock and poultry where swine are the primary reservoir [7]. The biotype distribution of *Y. enterocolitica* is different worldwide and also in the provinces of China. The predominant bioserotype in the world is 4/O: 3, especially in most European countries and Japan; while in America, bioserotype 1B/O: 8 strains once predominated, then substituted by serotype O: 3 [7]. The bioserotype distribution of *Y. enterocolitica* in China is different from other countries, predominantly 2/O: 9 and 3/O: 3, fewer 4/O: 3, and no pathogenic 1B/O: 8 strains [8].

Recently, various genotyping methods for *Y. enterocolitica* have been reported, e.g., restriction endonuclease analysis (REAC) [9], restriction fragment length polymorphism analysis (RFLP) [10], amplified fragment length polymorphism analysis (AFLP) [11], ribotyping [12], pulsed-field gel electrophoresis (PFGE) [13], multilocus sequence typing (MLST) [14], and multiple-locus variable-number tandem-repeat analysis (MLVA) [15], [16]. MLVA is widely used as a molecular genotyping method.

Multiple-locus VNTR analysis (MLVA) may use as few as two or dozens of VNTR loci for analysis to efficiently genotype many bacteria [17], including *Salmonella* [18], *Yersinia pestis* [19], *Mycobacterium tuberculosis* [20], *Escherichia coli O157:H7* [21], and *Legionella pneumophila* [22].

In 2007, Gierczynski et al. [15] reported the first *Y. enterocolitica* MLVA typing; the article analyzed the predominant bioserotype 4/O: 3 strains in Europe and North America. The study used the genome of *Y. enterocolitica* subsp. *palearctica* O: 3 strain Y11 and used six loci (of seven) for analysis. Our research was based on Gierczynski et al. method using seven VNTR loci with the same PCR primers to analyze pathogenic *Y. enterocolitica* in China. The purpose of our study was to collect MLVA types for pathogenic *Y. enterocolitica* in China; establish a database for MLVA typing; and compare our data to others.

## Results

### Simpson's index of diversity

The Simpson's index of diversity was calculated using BioNumerics software [23]. For the total strains, V5 exhibited the highest discriminatory power (DI = 90.43%), resolving 18 different alleles; the least variation was observed for locus V4. However, for the strains isolated from Ningxia province, V10 exhibited the highest discriminatory power (DI = 84.44%); while the most amount of alleles (14) was located in V5. The least variation also was located in the V4 locus. All of the VNTR loci showed high discriminatory power except for V4 (Table 1).

**Table 1:** The Simpson's index of diversity for the strains.

Locus	Period(bp)	total strains		Ningxia strains	
		numbers of alleles	simpson's index	numbers of alleles	simpson's index
V2A	6	17	61.83%	8	29.38%
V4	7	4	3.66%	1	0
V5	6	18	90.43%	14	83.70%
V6	6	14	85.36%	9	78.47%
V7	6	16	78.54%	9	67.32%
V9	12	6	72.65%	5	58.22%
V10	8	14	88.83%	12	84.44%

### MLVA analysis of all strains

**Figure 1:**
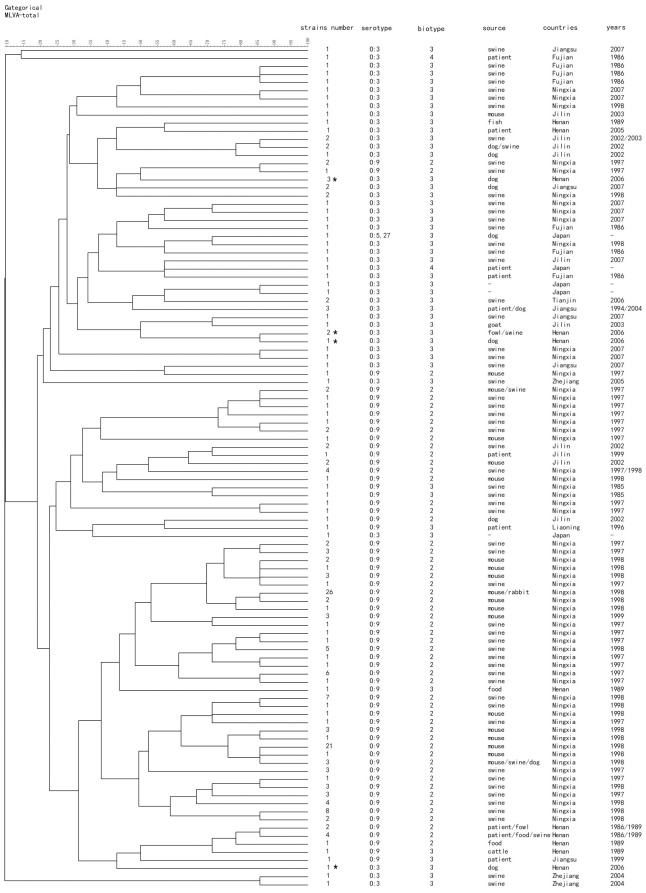
218 pathogenic *Y. enterocolitica* MLVA genotypes in this study * the isolates analyzed in **Figure 2**.

218 pathogenic *Y. enterocolitica* were divided into 104 MLVA genotypes (Fig. 1). We found strains that were isolated from the same province and same year where different reservoirs were classified to one MLVA genotype; this suggests an epidemiological link (especially for the Ningxia strains from 1997 to 1999). There was a correlation between the MLVA genotypes and serotypes. Both O: 3 and O: 9 serotype strains (except for a few) were separated into two MLVA genotyping clusters. O: 9 strains were primarily found in Ningxia, Jilin, and Henan provinces; while, the O: 3 strains were diversely distributed in different provinces in China. Four of the five strains isolated from Japan were classified to O: 3 serotype cluster according to their MLVA genotypes. This was in accordance with the serotype characteristic in China.

**Figure 2:**
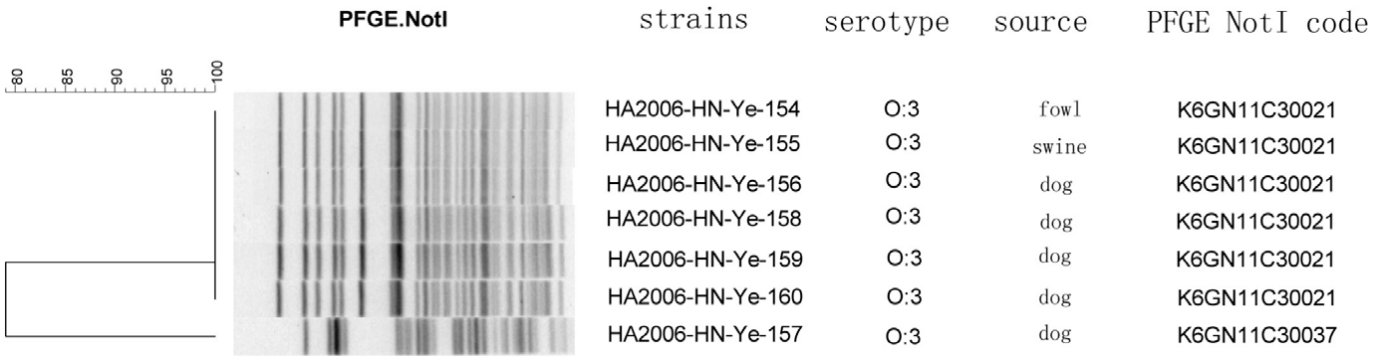
PFGE result of the strains isolated from Henan province in 2006.

The strains isolated from a patient in Jiangsu Province in 1994 had the same MLVA genotype as the strains isolated from dogs in 2004 where we previously showed this linkage [24]. The patient's strain had the same PFGE profile (*K6GN11C30021* PFGE-*Not*I profile) as the strains isolated from dogs in 2004 and 2007; however, here we showed that the isolates from patient and dogs in 2004 had the same MLVA genotype, while the strains isolated from dogs in 2007 displayed the different MLVA genotype. Therefore, MLVA may have a higher discriminatory power than PFGE. We also compared the MLVA and PFGE results of the seven bioserotype 3/O: 3 isolates from Henan province in 2006 and showed four MLVA genotypes and two PFGE *Not*I profiles (Fig. 2). The asterisks marked in figure 1 were analyzed strains in figure 2.

### MLVA analysis of the Ningxia strains

**Figure 3:**
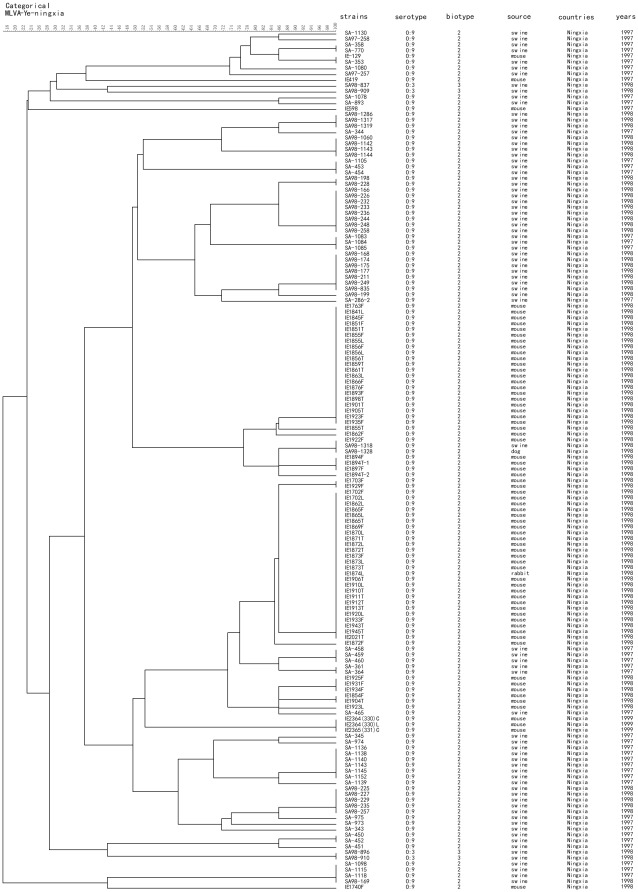
Cluster analysis of 148 pathogenic *Y. enterocolitica* in Ningxia province.

**Figure 4:**
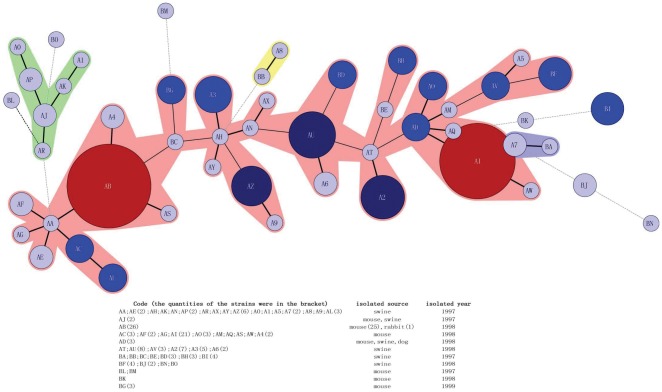
Minimum spanning tree of the 148 pathogenic *Y. enterocolitica*.

Among the total strains, 148 pathogenic *Y. enterocolitica* were isolated from Ningxia Province during 1997 to 1999. MLVA was able to distinguish 51 genotypes among the 148 pathogenic *Y. enterocolitica* (Fig. 3). All of the strains were bioserotype 2/O: 9 (except four bioserotype 3/O: 3). MLVA genotyping was able to identify strains isolated from the same source, especially for swine (SA98-1286 to SA-286-2) and mouse (IE1763F to IE1872F). Different MLVA genotypes were observed for strains isolated from the same source during different years, which shows the high discriminatory power of MLVA. We constructed a minimum spanning tree (MST) using MLVA data (Fig. 4). Most bioserotype 2/O: 9 strains were clustered in a large MLVA genotyping group (pink area) where only a few 3/O: 3 (BJ and BN) were distant from the center. Two cluster groups were observed among the strains, one was AB and AI (red area) for the strains isolated from mouse in 1998; another was AZ, AU and A2 (blue area) for the strains isolated from swine during 1997 to 1998.

### MLVA analysis based on six VNTR loci

In Europe, MLVA with six VNTRs markers was used to genotype *Y. enterocolitica* ssp. *palearctica* [15], [16]. This assay distinguished outbreak-related and sporadic isolates. To compare the genetic diversity of *Y. enterocolitica* in China and Europe, we analyzed the data with six VNTR loci. 90 MLVA6 genotypes were distinguished among 218 pathogenic *Y. enterocolitica*. O: 3 and O: 9 serotype strains were also separated into two MLVA6 genotyping clusters. O: 9 strains were mainly found in Ningxia, Jilin, and Henan provinces; while, the O: 3 strains displayed genotype heterogeneity. However, the isolates from the same province were clustered to same MLVA6 genotype for all the strains, which showed the relatedness with geographic distribution, especially for Ningxia province. The cluster results based on six VNTR loci were almost identical to MLVA genotype distribution applied seven VNTR loci for pathogenic *Y. enterocolitica* in China.

### Analysis of sequences flanking tandem repeats

We analyzed the sequences flanking tandem repeats for all the Ningxia isolated strains. From the beginning of the V2A locus we sequenced 76 bp, showing substitution in the 66 bp position with three O: 3 strains; from the tail of V2A locus we sequenced 126 bp, showing substitutions in the 9, 84 and 120 bp positions. From the beginning of the V4 locus we sequenced 72 bp, showing substitution in 4–6, 13, 14, 16, 29, 39–41 bp positions; from the tail of the V4 locus we sequenced 28 bp, and all the sequences were identical. From the beginning of the V5 locus we sequenced 122 bp and from the tail we sequenced 48 bp; the beginning of the V6 locus sequenced 86 bp and the tail 71 bp; the beginning of V9 locus sequenced 39 bp, the tail 40 bp; the beginning of V10 locus sequenced 41 bp, the tail 38 bp; all these loci showing large base substitution. There was no obvious regularity for the sequences flanking tandem repeats.

### MLVA analysis of highly pathogenic and nonpathogenic *Y. enterocolitica*

We performed the same analysis using bioserotype 1B/O: 8 strains from abroad and 19 nonpathogenic strains isolated from China. The data showed only V2A, V4 and V5 loci amplified the differential straps for these strains; whereas V6, V7, V9 and V10 loci showed no results.

## Discussion

In Gierczynski et al. research [15], MLVA based on six loci was able to distinguish 76 genotypes among 91 *Y. enterocolitica* isolated from worldwide origins and 41 genotypes among 51 non-epidemiologically linked bioserotype 4/O: 3 isolates. Only a slight correlation comparing the MLVA genotypes with geographic distribution of the isolates and a minor correlation comparing the MLVA genotypes with serogroups were observed in their study. Our research was based on continuous surveillance of *Y. enterocolitica* in China over a long period where we considered the similarity of the isolates from the same province. We included the V10 locus in our analysis and distinguished 104 genotypes among 213 pathogenic *Y. enterocolitica* isolated from China and five isolates from Japan. Most of the isolates were bioserotype 2/O: 9 and 3/O: 3 where the O: 3 and O: 9 serogroups were separated using MLVA genotyping.

This was an important difference between MLVA-genotypes distribution of *Y. enterocolitica* subsp. *palearctica* in China and Europe. The majority of strains tested in Europe belonged to unique genotype, few strains with no known epidemiological link belonged to the same MLVA genotype, such strains were isolated within the same country (Hungary, Poland, and Germany) in the reference “(15)”; while, our results indicated that the isolates from the same province of China were clustered to the same MLVA6 genotype, which showed the relatedness with geographic distribution. There was also correlation between the MLVA6 genotypes and serogroups in our research. The limited genetic diversity of MLVA results possibly reflected the recent dissemination (in evolutionary scale) of specific clones of *Y. enterocolitica* 2/O: 9 and 3/O: 3 strains in China.

We also performed the MLVA analysis for the highly pathogenic strains from abroad and 19 nonpathogenic *Y. enterocolitica* isolated in China where only V2A, V4, and V5 loci showed differential results. Gulati et al. [25] screened eight VNTR loci and used four loci to generate 26 MLVA genotypes among 81 strains of *Y. enterocolitica* biovar 1A, based on the whole genome sequence of the highly pathogenic bioserotype 1B/O: 8 *Y. enterocolitica* 8081 in 2009. Only four loci could be amplified from the screened eight VNTR loci for *Y. enterocolitica* biovar 1A strains. Our research also showed only three loci could be amplified for highly and nonpathogenic *Y. enterocolitica* based on the genome of bioserotype 4/O: 3 *Y. enterocolitica* Y11. In Gierczynski et al. study, six highly pathogenic *Y. enterocolitica* 1B/O: 8 strains could not be genotyped by MLVA, which was in accordance with our results. Because no highly pathogenic bioserotype 1B/O: 8 was isolated from China, we performed the MLVA analysis for *Y. enterocolitica* ssp. *palearctica* in China and established a database to provide epidemiological information for *Y. enterocolitica* outbreaks in the future.

The distribution, characters, and functions of seven VNTR loci used in our study were not described in the reference “(15)”, therefore we analyzed the seven VNTR loci using Blastn and Blastp searches in the NCBI data base. The results show V2A is a sensory histidine kinase; V4 is a putative glycerate kinase; V5 is a putative hydrogenase isoenzymes formation protein; V6 is a hypothetical protein; V7 is a putative GTP-binding protein; V9 is a transcriptional regulator; and V10 is a hypothetical protein. These loci were generally located in the open reading frame of structural genes. V2A, V4 and V5 loci shared homology with the highly pathogenic bioserotype 1B/O: 8 strain 8081 through blast results; and may explain the reason for the amplifications. However, V6, V7, V9 and V10 loci only exist in weakly pathogenic *Y. enterocolitica* strains, as confirmed herein.

## Materials and Methods

### Bacterial strains

A total of 218 pathogenic *Y. enterocolitica* strains were used where the number of isolates from patients was 11 and all the isolates were from sporadic yersiniosis cases. 52 strains were bioserotype 3/O: 3; two were 4/O: 3, one was 3/O: 5,27, 157 were 2/O: 9, and six were 3/O: 9. During the 1997 to 1998, we performed a large scale of surveillance for animal reservoirs of *Y. enterocolitica* in Ningxia province, and isolated lots of pathogenic *Y. enterocolitica* bioserotype 2/O: 9 strains from mouse and swine. 213 strains were isolated from China and five strains were provided by Dr. H. Fukushima. All of the strains were PCR tested to detect virulence genes (Table 2).

**Table 2:** The pathogenic *Y. enteroclitica* strains used in this study.

Strains number	ST	BT	Source	Country	Year	PCR results					
						*ail*	*ystA*	*ystB*	*yadA*	*virF*	*rfbc*
1	O:3	3	patient	Fujian	1986	+	+	-	−	−	+
5(1)	O:3	3	swine	Fujian	1986	+	+	-	+	+	+
1	O:3	4	patient	Fujian	1986	+	+	-	−	−	+
1	O:3	3	Fish	Henan	1989	+	+	-	+	+	+
5(3)	O:3	3	Dog	Henan	2006	+	+	-	+	+	+
1	O:3	3	fowl	Henan	2006	+	+	-	+	+	+
1	O:3	3	swine	Henan	2006	+	+	-	−	−	+
1	O:3	3	patient	Henan	2005	+	+	-	+	+	+
3(2)	O:9	2	patient	Henan	1986	+	+	-	+	+	−
1	O:9	3	cattle	Henan	1989	+	+	-	−	−	−
1	O:9	3	food	Henan	1989	+	+	-	+	+	−
1	O:9	2	fowl	Henan	1989	+	+	-	−	−	−
1	O:9	2	swine	Henan	1989	+	+	-	−	−	−
2	O:9	2	food	Henan	1989	+	+	-	+	+	−
1	O:3	3	patient	Jiangsu	1994	+	+	-	+	+	+
2	O:3	3	Dog	Jiangsu	2004	+	+	-	+	+	+
2(1)	O:3	3	Dog	Jiangsu	2007	+	+	-	+	+	+
2	O:3	3	swine	Jiangsu	2007	+	+	-	+	+	+
1	O:3	3	swine	Jiangsu	2007	+	+	-	−	−	+
1	O:9	3	patient	Jiangsu	1999	+	+	-	−	−	−
2	O:3	3	Dog	Jilin	2002	+	+	-	+	+	+
2	O:3	3	swine	Jilin	2002	+	+	-	+	+	+
1	O:3	3	mouse	Jilin	2003	+	+	-	+	+	+
1	O:3	3	swine	Jilin	2003	+	+	-	+	+	+
1	O:3	3	goat	Jilin	2003	+	+	-	+	+	+
1	O:3	3	swine	Jilin	2007	+	+	-	+	+	+
1	O:9	2	patient	Jilin	1999	+	+	-	−	−	−
1	O:9	2	Dog	Jilin	2002	+	+	-	+	+	−
2	O:9	2	mouse	Jilin	2002	+	+	-	+	+	−
2	O:9	2	swine	Jilin	2002	+	+	-	+	+	−
1	O:9	3	patient	Liaoning	1996	+	+	-	+	+	−
4	O:3	3	swine	Ningxia	1998	+	+	-	+	+	+
7(1)	O:3	3	swine	Ningxia	2007	+	+	-	+	+	+
3	O:9	2	mouse	Ningxia	1997	+	+	-	+	+	−
41	O:9	2	swine	Ningxia	1997	+	+	-	+	+	−
63	O:9	2	mouse	Ningxia	1998	+	+	-	+	+	−
1	O:9	2	rabbit	Ningxia	1998	+	+	-	+	+	−
1	O:9	2	Dog	Ningxia	1998	+	+	-	+	+	−
32	O:9	2	swine	Ningxia	1998	+	+	-	+	+	−
3	O:9	2	mouse	Ningxia	1999	+	+	-	+	+	−
2	O:9	3	swine	Ningxia	1985	+	+	-	+	+	+
3	O:3	3	swine	Tianjin	2006	+	+	-	+	+	+
1	O:3	3	patient	Tianjin	2006	+	+	-	+	+	+
2(1)	O:3	3	swine	Zhejiang	2004	+	+	-	+	+	+
1	O:3	3	swine	Zhejiang	2005	+	+	-	−	−	+
*Pa40134	O:3	4	patient	Japan	-	+	+	-	+	+	+
*ye3vp−/03	O:3	3	-	Japan	-	+	+	-	−	−	−
*ye3vp5/03	O:3	3	-	Japan	-	+	+	-	+	+	+
*D92	O:5,27	3	Dog	Japan	-	+	+	-	+	+	−
*ye4/03	O:3	3	-	Japan	-	+	+	-	+	+	+

Note: The bracket next to strain numbers are the strains that have lost the plasmid;

* strains from Japan; ST, serotype; and BT, biotype; *ail*, attachment invasion locus of *Y. enterocolitica*; *ystA*, the production of heat-stable enterotoxin in *Y. enterocolitica*; *ystB*, encode an enterotoxin present mainly in biotype 1A strains; *yadA*, the plasmid-borne virulence gene involved in autoagglutination, serum resistance, and adhesion; *virF*, encode transcriptional activators of the *yop* regulon in pYV plasmid; *rfbc*: located in the *rfb* gene cluster, responsible for the biosynthesis of the O side chain of *Y. enterocolitica* O: 3 strain.

We selected three highly pathogenic foreign strains and compared them to 19 non-pathogenic strains isolated in China (Table 3).

**Table 3:** Highly and nonpathogenic *Y. enterocolitica* strains used in this study.

Strains	ST	BT	Source	Country	Year	PCR results					
						*ail*	*ystA*	*ystB*	*yadA*	*virF*	*rfbc*
WA	O:8	1B	-	America	-	+	+	−	+	+	-
Pa12986	O:8	1B	patient	Japan	-	+	+	−	+	+	-
YE92010	O:8	1B	patient	Japan	-	+	+	−	+	+	-
ZJ06LQ58	undetermined	1A	mouse	Jiangsu	2006	−	−	+	−	−	-
Y40	O:8	1A	swine	Jiangsu	1986	−	−	+	−	−	-
yang4	O:8	1A	sheep	Jiangsu	2005	−	−	+	−	−	-
Y20	O:8	1A	swine	Jiangsu	1986	−	−	+	−	−	-
Y23	O:8	1A	swine	Jiangsu	1986	−	−	+	−	−	-
948	O:8	-	food	Henan	2005	−	−	-	−	−	-
HNY042	O:8	1A	cattle	Henan	2005	−	−	+	−	−	-
HNY043	O:8	1A	cattle	Henan	2005	−	−	+	−	−	-
sui35	undetermined	5	sheep	Henan	2005	−	−	-	−	−	-
200512189	O:8	1A	food	Shenzhen	2005	−	−	+	−	−	-
02-162	O:6,30	1A	patient	Shandong	1986	−	−	+	−	−	-
02-157	O:5	1A	patient	Shandong	1986	−	−	-	−	−	-
02-159	O:6,30	1A	patient	Shandong	1986	−	−	+	−	−	-
02-160	O:6,30	1A	patient	Shandong	1986	−	−	+	−	−	-
02-144	O:5	1A	patient	Shandong	1986	−	−	+	−	−	-
02-148	O:5	1A	patient	Shandong	1986	−	−	+	−	−	-
02-152	O:5	1A	patient	Shandong	1986	−	−	+	−	−	-
02-143	O:5	1A	patient	Shandong	1986	−	−	+	−	−	-
GM234	undetermined	1A	food	Shandong	2007	−	−	+	−	−	-

### DNA extraction

The bacteria were inoculated on BHI agar; incubated for 24 hours at 25°C; and the genome DNA of the bacteria were extracted using a DNeasy tissue kit (Qingen, Germany) using the manufacturer's instructions.

### PCR analysis and sequencing

Using the primers from the reference method [15], VNTR loci were amplified using the primers at a concentration of 0.2 uM with Taq DNA polymerase (TaKaRa, Japan) in the reaction buffer supplied by the manufacturer in a 20-ul reaction volume. PCR amplifications were performed: initial denaturation at 94°C for 10 min; followed by 35 cycles: denaturation at 94°C for 30 s, annealing at 58°C for 30 s, and extension at 72°C for 30 s; and a final extension at 72°C for 3 min. All of the PCR products were sequenced (TaKaRa); and the fragments of each sequence were merged and aligned using DNAstar software; then we computed the numbers of tandem repeats for each strain.

### Cluster analysis

Clustering of the MLVA types was performed using BioNumerics software (ver. 5.10) with categorical coefficient of similarity and UPGMA (the un-weighted pair group method using the arithmetic mean) method to estimate genetic differences. For the higher proportion (67.89%) of the isolated strains found in Ningxia province from 1997 to 1999, we tested induplicate. Then, we analyzed the sequences before or after the tandem repeats for each strain, intended.
